# Trajectories and management of vascular risk following the diagnosis of multiple sclerosis: A population-based matched cohort study between 1987 and 2018 in England

**DOI:** 10.1177/13524585241287388

**Published:** 2024-10-17

**Authors:** Raffaele Palladino, Jeremy Chataway, Mekha Mathew, Azeem Majeed, Ruth Ann Marrie

**Affiliations:** Department of Primary Care and Public Health, School of Public Health, Imperial College London, London, UK; Department of Public Health, University of Naples Federico II, Naples, Italy; Queen Square Multiple Sclerosis Centre, Department of Neuroinflammation, UCL Queen Square Institute of Neurology, Faculty of Brain Sciences, University College London, London, UK; University College London Hospitals Biomedical Research Centre, National Institute for Health Research, London, UK; Department of Primary Care and Public Health, School of Public Health, Imperial College London, London, UK; Department of Primary Care and Public Health, School of Public Health, Imperial College London, London, UK; Departments of Medicine and Community Health Sciences, Max Rady College of Medicine, Rady Faculty of Health Sciences, University of Manitoba, Winnipeg, MB, Canada

**Keywords:** Multiple sclerosis, epidemiology, vascular management, diabetes, hypertension, body mass index

## Abstract

**Background:**

People with multiple sclerosis (PwMS) have an increased cardiovascular and cerebrovascular disease burden, but this could be mitigated by vascular risk factor management.

**Objectives:**

We compared the trajectories of vascular risk factors, vascular comorbidities and clinical management in PwMS against the general population post-MS diagnosis while controlling for frailty.

**Methods:**

Retrospective longitudinal analysis using English data from the Clinical Practice Research Datalink between 1987 and 2018 comprising PwMS matched with up to six controls without MS by age, sex and general practice.

**Results:**

We compared 12,251 PwMS with 72,572 matched controls; 3.8% of PwMS had mild–moderate frailty, 1.2% more than matched controls. Compared to controls, PwMS had an elevated incidence of Type 2 diabetes (HR 1.18, 95% CI (1.04, 1.34)), and starting antihypertensive medications (HR 1.40, 95% CI (1.33, 1.47)). Among those with hypertension at baseline, blood pressure trajectories did not differ between PwMS and controls. PwMS had increased rates of meeting targets for hypertension management (HR 1.25, 95% CI (1.12, 1.41)).

**Conclusion:**

The observation that PwMS with hypertension are more likely to meet treatment targets than matched controls is encouraging, but the elevated rates of vascular comorbidities suggest that tighter vascular management may be needed in this population.

## Introduction

People with Multiple Sclerosis (PwMS) have a 30% increased prevalence of macrovascular disease compared with people without MS, partially explained by the elevated prevalence of vascular risk factors and comorbidities including diabetes, hypertension and dyslipidemia.^1–4^ These factors are associated with increased relapse rate, disability progression and reduced health-related quality of life.^2,5,6^

Vascular comorbidities are potentially preventable and modifiable.^7,8^ At the time of MS diagnosis, we found that PwMS had an increased prevalence of diabetes and hypertension than controls, but paradoxically, a *decreased* likelihood of being treated for those conditions.^9^ However, a Canadian study suggested that PwMS were as or more likely to meet treatment targets for hypertension and diabetes post-diagnosis of MS.^2^ Longitudinal studies evaluating the post-diagnosis evolution of vascular clinical management are lacking.

Frailty reflects a decline in biological reserves across various organ systems and an increased susceptibility to physiological decompensation following exposure to stressors.^10^ It can be considered as a measure of accumulation of health deficits and is associated with increased macrovascular disease risk and mortality.^11–13^ A higher percentage of PwMS is frail than people from the general population,^9,14^ and frailty is associated with disability measured using the Expanded Disability Status Scale (EDSS).^14,15^

We compared the trajectories of vascular risk factors, vascular comorbidities and the clinical management in PwMS and the general population, following the diagnosis of MS and accounting for frailty. We used representative data from the English population to extract information on PwMS and matched controls from 1987 to 2018.^16^

## Methods

### Study design

We conducted a retrospective longitudinal analysis using the Clinical Practice Research Datalink (CPRD). We included people with a diagnosis of MS between 1 January 1987 and 30 September 2018 and matched with up to six controls. The study follow-up period ended on 30 September 2018. Ethical approval was obtained from the CPRD Independent Scientific Advisory Committee (protocol no. 18_279R).

### Data source

The CPRD^16^ is managed by the UK Department of Health and provides de-identified data from patients registered with any practice in the a network of UK primary care practices since January 1987.^17^ For the time frame of this study, the CPRD GOLD data source, from which this database was sampled, covered 7% of the UK population and was broadly representative regarding age, sex and ethnicity.^18,19^ The pay-for-performance targets to incentivize chronic disease management results in well-maintained disease registries, ensuring high-quality data.^20^ Consistent with previous research,^9,21^ to reduce the risk of ascertainment and selection bias, we restricted our data only to those living in England, as linked mortality data from the Office for National Statistics (ONS) and secondary care data from Hospital Episode Statistics (HES) were available for this subset of CPRD.^16^

### Study population

The study population has been described previously.^1,21^ Briefly, we identified possible MS cases based on patient’s electronic healthcare records, coded using READ code system (primary care), ICD-X codes (secondary care) and British National Formulary (BNF) codes (prescription of MS disease-modifying therapies). We adopted a case-finding algorithm to identify PwMS based on the presence of ⩾ 3 MS events recorded in the patient’s available clinical history, where the first MS diagnosis code was assigned as the index date.^1^ This algorithm has been widely used in previous research using CPRD data,^1,9,21^ recently validated using a similar dataset in the United Kingdom, and consistent with validated algorithms in other countries.^22^ Consistent with previous studies,^1,21,22^ the PwMS cohort met these criteria: (1) diagnosis after 1 January 1987 (magnetic resonance imaging (MRI) availability); (2) ⩾ 1 year of continuous registration with a CPRD practice before initial MS event; (3) gender-defined (male or female); (4) applicable birthdate; (5) age ⩾ 18 years at cohort entry; (6) MS events recorded before the date of death; and (7) validity of patients’ clinical records in terms of continuous follow-up (corresponding to the CPRD definition of up-to-standard (UTS)).^1^

A randomly matched control cohort of to up to six people without MS by age, sex and general practice was selected; six controls were chosen to reduce variance.^23^ For the controls, additional selection criteria were as follows: (1) UTS clinical data recorded during the study period; (2) no MS or other demyelinating disease event (acute disseminated encephalomyelitis, optic neuritis, transverse myelitis and central nervous system demyelination) recording; (3) validity of reference cohorts’ clinical records in terms of continuous follow-up and (4) people who survived to the end of the study period were censored at the date of last data collection for the CPRD practice.

Individuals in both cohorts were followed from their index date (T0, earliest possible was 1987) until 2018, with maximum follow-up of 30 years.

### Study variables

Consistent with prior research based on CPRD data,^1,21^ we defined the study variables using a validated approach relying on comprehensive primary care code lists and ICD-X codes, and prescribing data based on BNF codes.^1,21^ Specifically, we created sociodemographic variables: age, gender (women/men), ethnicity (White/other) and index of multiple deprivation (quintiles).^24^ As risk-factor/clinical variables, we defined smoking status (current smoker/ former smoker/non-smoker), diagnosis of diabetes and hypertension, body mass index (BMI) and systolic and diastolic blood pressure (SBP and DBP). The prescription data included treatment with lipid-lowering, oral antidiabetic and antihypertensive.

To define frailty, we used the electronic frailty index (eFI), which encompasses 36 deficits and is widely adopted in the United Kingdom. For each individual, the sum of identified deficits was divided by the total number of deficits that the score includes. Individuals were classified as fit (a score < 0.12), mildly frail (0.12–0.24), moderately frail (0.24–0.36), or severely frail (⩾ 0.36).^25^ As a measure of total healthcare utilisation, we included the number of primary care visits, to account for differences in healthcare utilisation between the MS and matched cohorts (surveillance bias). Finally, we included a variable regarding whether the National Institute for Health and Care Excellence (NICE)^26^ defined target for blood pressure management (as a binary outcome) was met, calculated as blood pressure values lower than 140/90 mmHg in people with hypertension.

### Statistical analysis

We compared characteristics of PwMS and controls. Descriptive data were reported as mean ± SD, median (IQR) or percentage, as appropriate. Comparisons were assessed using chi-square tests for categorical variables, two-sample *t*-tests and Cohen’s *d* statistics for continuous variables.

Latest clinical data were obtained (within 5 years from index year) to reduce missing data at baseline.^1,21^ After checking multiple imputation assumptions, missing data for blood pressure (49.9%) and BMI (50%) at index year were handled through multiple imputation by chained equations (10 copies) and combined using the Rubin’s rules.

The exposure of interest was MS status (yes/no). Outcomes of interest were incidence of diabetes, hypertension, starting treatment for these conditions and rate of reaching NICE targets for hypertension treatment. Only conditions of interest diagnosed after the index year (year of MS diagnosis or matched index year for controls) were considered as incident cases, and people with a history of the relevant outcome at baseline (e.g. diabetes) were excluded from the analysis. We used the Nelson–Aalen cumulative hazard curves to plot the estimated incidence of each outcome. Using multivariable cox proportional hazard regression, we modelled differences in the hazard rates of each outcome. In cox models, individuals were considered at risk for the entire study period or until reaching the study endpoints and censored in case of death, transfer out of the general practice or end of study period. The proportional hazard assumption was met as assessed using plots of log(−log survival time) against log survival time and Schoenfeld residuals against survival time, and linear regression of Schoenfeld residuals on time to test for independence between residuals and time. When modelling differences in rates of achieving NICE targets for blood pressure management, we considered all available blood pressure measurements and indicated as date of target achievement the first time the measurement was kept within the targets.

To assess changes in vascular risk factor trajectories over the study period, mixed-effect linear regression models were employed to account for the presence of multiple records within each individual. For each outcome, we estimated the average difference over time and the slope yearly change by fitting an interaction term between the time and MS status.

All multivariable models were adjusted for gender (female as reference), ethnicity, region, deprivation index, number of primary care visits, smoking status, and eFI. In the longitudinal models, when applicable, variables were included as time-varying, accounting for the yearly change in the vascular risk factors (e.g. BMI, SBP and DBP), frailty status (eFI) and healthcare resource utilisation (number of primary care visits). We repeated these analyses after stratifying by sex to assess effect modification.

#### Sensitivity analysis

PwMS use more healthcare than the general population,^27–29^ which could influence vascular risk management. Hence, we conducted a sensitivity analysis stratifying our study population by tertiles of primary care visits in the year before the index year.

Model assumptions were tested using graphical methods. Results are presented as regression coefficients (coeff.), hazard ratios (HRs), which can be roughly interpreted as incident rate ratios in this context,^30^ and 95% confidence intervals (95% CI), as appropriate. A *p*-value less than 0.05 was considered statistically significant. We used Stata 17 MP (StataCorp. 2017, College Station, TX: StataCorp LLC) to conduct analyses.

## Results

The final cohort included 12,251 PwMS and 72,572 matched controls. Overall, 70% of the sample was female, the average age at index year was 44.9 years, and 20.4% of the sample lived in the most deprived areas. Nearly 4% of PwMS were mildly to moderately frail at the index date, on average, 1.2% more than matched controls. PwMS had a 70% increased standardised mean difference in primary care visits than controls (Table 1).

**Table 1.** Characteristics of the study population at index year.

	Male				Female				Overall			
	PwMS	Matched controls	Standardised mean difference	*p*-value	PwMS	Matched controls	Standardised mean difference	*p*-value	PwMS	Matched controls	Standardised mean difference	*p*-value
*N*	3685	21,931			8566	50,640			12,251	72,572		
Follow-up time (years)	9.9 (6.1)	11.4 (6.5)	−0.23 (−0.27, −0.20)	<0.001	10.4 (6.3)	11.5 (6.5)	−0.17 (−0.19, −0.15)	<0.001	10.3 (6.3)	11.5 (6.5)	−0.19 (−0.20, −0.17)	<0.001
Female, *N* (%)									8566 (69.9)	50,640 (69.8)		0.752
Age (years)	46.3 (13.3)	46.3 (13.3)	0 (−0.3, 0.03)	0.852	44.3 (13.3)	44.3 (13.3)	0 (−0.02, 0.02)	0.907	44.9 (13.3)	44.9 (13.3)	0 (−0.02, 0.02)	0.727
Ethnicity – White, *N* (%)	20,243 (92.3)	3444 (93.5)		0.013	46,341 (91.5)	8059 (94.1)		<0.001	11,503 (93.9)	66,585 (91.2)		<0.001
Smoking status, *N* (%)												
Non-smoker	1528 (41.5)	11,794 (53.8)		<0.001	4240 (49.5)	30,360 (60.0)		<0.001	5768 (47.1)	42,155 (58.1)		<0.001
Ex-smoker	644 (17.5)	3215 (14.7)			1191 (13.9)	5854 (11.6)			1835 (15)	9069 (12.5)		
Current smoker	1513 (41.1)	6923 (31.6)			3135 (36.6)	14,425 (28.5)			4648 (37.9)	21,348 (29.4)		
eFI ratio	0.02 (0.04)	0.01 (0.03)	0.32 (0.28, 0.35)	<0.001	0.03 (0.04)	0.02 (0.04)	0.25 (0.23, 0.27)	<0.001	0.03 (0.04)	0.02 (0.04)	0.25 (0.23, 0.27)	<0.001
Fit, *N* (%)	3582 (97.2)	21,564 (98.3)		<0.001	8205 (95.8)	49,149 (97.1)		<0.001	11,787 (96.2)	70,714 (97.4)		<0.001
Mild frailty, *N* (%)	102 (2.8)	359 (1.6)			345 (4.0)	1430 (2.8)			447 (3.7)	1789 (2.5)		
Moderate frailty, *N* (%)	9 (0.0)	9 (0.0)			16 (0.2)	59 (0.1)			17 (0.1)	68 (0.1)		
Severe frailty, *N* (%)	0 (0.0)	0 (0.0)			0 (0.0)	1 (0.0)			0 (0.0)	1 (0.0)		
Number of primary care visits in previous year	6.9 (10.3)	2.2 (5.0)	0.78 (0.74, 0.81)	<0.001	8.2 (11.5)	3.2 (6.1)	0.7 (0.68, 0.72)	<0.001	7.8 (11.2)	2.9 (5.9)	0.71 (0.69, 0.73)	<0.001
Index of multiple deprivation (IMD), *N* (%)												
1Q – least-deprived	505 (13.7)	3012 (13.7)		1.000	1254 (14.6)	7409 (14.6)		1.000	1759 (14.4)	10,421(14.4)		1.000
2Q	681 (18.5)	4040 (18.4)			1588 (18.5)	9359 (18.5)			2268 (18.5)	13,399 (18.5)		
3Q	649 (17.6)	3863 (17.6)			1532 (17.9)	9061 (17.9)			2181 (17.8)	12,924 (17.8)		
4Q	747 (20.2)	4439 (20.3)			1610 (18.8)	9514 (18.8)			2357 (19.2)	13,953 (19.2)		
5Q – most deprived	759 (20.6)	4526 (20.6)			1739 (20.3)	10,287 (20.3)			2498 (20.4)	14,813 (20.4)		
Missing data	344 (9.3)	2052 (9.4)			843 (9.8)	5009 (9.9)			1187 (9.7)	7062 (9.7)		

PwMS: People with Multiple Sclerosis.

Year of MS diagnosis or matched year for controls was considered as index year. Individuals were classified as fit if the eFI score was below 0.12, mildly frail if the score was between 0.12 and 0.24, moderately frail if the score was between 0.24 and 0.36, and severely frail if the score was 0.36 and above. Chi-square and Student’s *t*-test were employed to assess differences in variables distribution between people with MS and matched controls, as appropriate, while Cohen’s *d* was computed to calculate the standardised mean difference for continuous variables between groups. For the interpretation of the Cohen’s *d*, 0.2, 0.5, and 0.8 are thresholds generally considered for small, moderate, and large effect, respectively. Differences in diagnoses and commencement of vascular treatment over time.

All incidence rates herein are per 100,000 person-years. Following an MS diagnosis, the 10-year incidence rate of Type 2 diabetes was 292.0 (257.9–330.7) in PwMS and 184.6 (173.5–196.3) in matched controls (Supplementary Table 1; Supplementary Figure 1). In men with MS, the 10-year incidence rate was 297.9 (236.8–374.7) and in matched controls, it was 218.5 (196.9–242.5), while in women with MS, it was 289.7 (249.8–335.9) and in matched controls, it was 170.3 (157.8–183.8; Supplementary Table 1). Over the study period, on multivariable analysis, PwMS had an 18% higher rate of incident Type 2 diabetes than matched controls (HR 1.18, 95% CI (1.04, 1.34)). Differences were greater when restricting analyses to women (HR 1.28, 95% CI (1.10, 1.48)); no difference was found in men (HR 0.99, 95% CI (0.79, 1.24)). PwMS had 23% increased rates of starting antidiabetic medications, as compared with matched controls. Sex-stratified results were similar (overall: HR 1.40, 95% CI (1.33, 1.47); women: HR 1.46, 95% CI (1.36, 1.55); men: HR 1.27, 95% CI (1.16, 1.40); Supplementary Figure 2).

**Figure 1.**
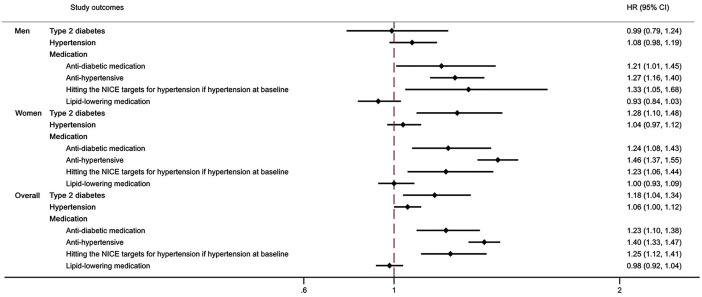
Differences in the rate of developing Type 2 diabetes and in the rates of vascular risk medication initiation or reaching NICE target between PWMS and controls, years 1987–2019. PwMS: People with Multiple Sclerosis; HR: hazard ratio; NICE: National Institute for Health and Care Excellence. Adjusted hazard ratios between PwMS and matched controls were estimated employing Cox Proportional Hazard regression models. Models were adjusted for gender, age, ethnicity (White/non-White), deprivation, smoking status, BMI, systolic and diastolic blood pressure, eFI ratio, number of primary care visits in the year before, and year.

The 10-year incidence rate of hypertension was 1433.3 (1352.5–1518.9) in PwMS post-MS diagnosis and 1210.3 (1180.7–1240.7) in matched controls. In men with MS, the hypertension incidence rate was 1711.4 (1549.3–1890.5) and in matched controls, it was 1445.2 (1386.2–1506.7). In women with MS, it was 1322.7 (1231.6–1420.5) and in matched controls, it was 1111.5 (1077.7–1146.3) (Supplementary Table 1; Supplementary Figure 3). On multivariable analysis, no statistically significant differences were found in the rate of incident hypertension over time between PwMS and matched controls. However, PwMS had 40% increased rates of starting antihypertensive medications (HR 1.40, 95% CI (1.33, 1.47); Supplementary Figure 4). Furthermore, among those with hypertension at baseline, PwMS had 25% increased rates of hitting NICE targets for hypertension management (HR 1.25, 95% CI (1.12, 1.41)). Sex-stratified results were similar (Figure 1; Supplementary Table 2; Supplementary Figure 5).

No differences were observed in the rates of starting lipid-lowering medications over time between PwMS and matched controls on multivariable analysis (Figure 1; Supplementary Figure 6).

### Differences in risk factor trajectory over time

**Figure 2.**
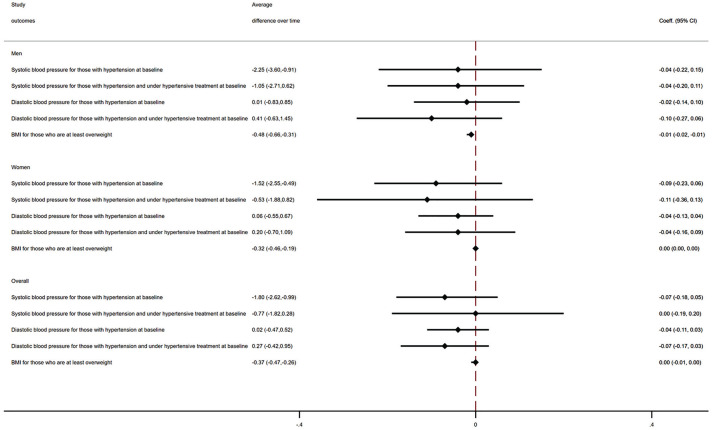
Differences in BMI and blood pressure values between PwMS and matched controls, years 1987–2019. PwMS: People with Multiple Sclerosis; Coeff: regression coefficients. Mixed-effects linear regression models were employed to account for the hierarchical structure of the data (multiple records within each individual). For each outcome, we estimated the average difference over time and the slope yearly change (by fitting an interaction term between time and MS status). Models were adjusted for gender, age, ethnicity (White/non-White), deprivation, smoking status, BMI, systolic and diastolic blood pressure, eFI ratio, number of primary care visits in the year before the diagnosis, and year.

Over the entire study period, PwMS who had hypertension at baseline had on average a 1.8 mmHg lower systolic blood pressure than matched controls (coeff. −1.80, 95% CI (−2.62, −0.99)) (Figure 2). No yearly slope change over the study period was found. Findings were consistent when analyses were stratified by sex. No differences were observed when assessing changes in diastolic blood pressure for those with a diagnosis of hypertension at index year. For those who were at least overweight at baseline, PwMS had on average a 0.4 lower BMI (coeff. −0.37, 95% CI (−0.47, −0.26)). However, no clinically meaningful differences were observed in yearly slope changes over the study period. Findings were similar in sex-stratified analyses.

### Sensitivity analysis

No clinically meaningful differences were observed in vascular risk factor trajectories in high-risk subgroups when stratifying analyses by tertiles of primary care utilisation in the year before the index year (Table 2).

**Table 2.** Differences in vascular risk factor management by tertile of primary care visits in the year before baseline between PwMS and controls, years 1987–2019.

	Lower tertile			Medium tertile			Higher tertile		
	PwMS = 3097			PwMS = 10,778			PwMS = 20,608		
	MC = 41,186			MC = 2929			MC = 6225		
	Coeff.	95% CI		Coeff.	95% CI		Coeff.	95% CI	
Blood pressure									
Systolic blood pressure									
Average difference over time	−1.90	−3.84	0.05	**−3.40**	**−5.14**	**−1.66**	**−1.34**	**−2.35**	**−0.34**
Yearly slope change	0.08	−0.19	0.36	−0.21	−0.44	0.01	0.00	−0.15	0.15
Systolic blood pressure for those with hypertension and under antihypertensive treatment at baseline									
Average difference over time	**−6.73**	**−12.65**	**−0.82**	−0.35	−2.87	2.17	−0.76	−1.95	0.44
Yearly slope change	0.47	−0.56	1.50	−0.35	−0.70	0.00	0.00	−0.18	0.17
Diastolic blood pressure									
Average difference over time	−0.21	−1.29	0.86	−0.67	−1.76	0.42	0.23	−0.41	0.87
Yearly slope change	0.03	−0.10	0.17	−0.05	−0.20	0.09	−0.01	−0.10	0.08
Diastolic blood pressure for those with hypertension and under antihypertensive treatment at baseline									
Average difference over time	−1.08	−5.12	2.97	0.77	−0.96	2.49	0.24	−0.53	1.02
Yearly slope change	0.29	−0.39	0.98	−0.21	−0.45	0.02	−0.05	−0.16	0.06
BMI									
For those who are at least overweight									
Average difference over time	**−0.38**	**−0.52**	**−0.24**	−0.04	−0.27	0.19	**−0.45**	**−0.64**	**−0.26**
Yearly slope change	**−0.02**	**−0.02**	**−0.01**	**0.00**	**0.00**	**0.01**	**−0.02**	**−0.02**	**−0.01**

PwMS: People with Multiple Sclerosis; MC: matched controls; Coeff: regression coefficient.

Mixed-effects linear regression models were employed to account for the hierarchical structure of the data (multiple records within each individual). For each outcome, we estimated the average difference over time and the slope yearly change (by fitting an interaction term between time and MS status). Models were adjusted for gender, age, ethnicity (White/non-White), deprivation, smoking status, BMI, systolic and diastolic blood pressure, eFI ratio, and year and stratified by tertiles of primary care visits in the year before baseline. In the second row, the sample size in each tertile is reported.

In bold results with p-value < 0.05.

## Discussion

This large population-based matched cohort study included 12,251 PwMS and 72,572 matched controls followed for up to 30 years. Following the diagnosis of MS, PwMS had an increased rate of Type 2 diabetes, but not hypertension. Following the diagnosis, PwMS were more likely to start the treatment for vascular disease than matched controls, even after controlling for confounders, including frailty and healthcare use. PwMS had 23% higher rates of commencing antidiabetic medication, and 40% higher rates of commencing antihypertensive medication accompanied by a 25% higher rate of achieving NICE targets. However, the increased rates in commencing antihypertensive medication for PwMS did not translate to differences in blood pressure values over time, as compared with matched controls. Rates of commencing lipid-lowering medication did not differ between PwMS and matched controls.

These findings contrast with our findings at *baseline* (diagnosis) where, the vascular risk for PwMS was greater (30% diabetes and 6% hypertension) but the vascular management was worse than the matched controls, with a 56% lower probability of initiating antidiabetic medication and 66% lower probability of commencing antihypertensive medication.^9^ This study demonstrates an increased intensity of vascular risk management after diagnosis (by 20%–40%) for PwMS, which might partially explain the higher rate of achieving NICE targets. The increased intensity of vascular management might also include people at high risk of vascular disease. This might, possibly, explain the similar incidence of hypertension between PwMS and matched controls in adjusted analysis. Our findings were similar to those in a prior Canadian study, one of the few studies to examine hypertension incidence in PwMS.^2^ Most prior studies examined prevalence which has been elevated in PwMS.^3^ These apparently contradictory findings may reflex complex evolution of vascular risk by age and disease course and warrant further evaluation.

Overall, the tightened vascular management did not translate into sustained vascular risk reduction over the study period as evidenced by the lack of reduction in blood pressure values compared to controls. This implies that management is still suboptimal and that further approaches are needed.

Our findings are consistent with an Italian study which reported increased frequency of Type 2 diabetes and increased likelihood of antihypertensive medication in PwMS as compared with matched controls.^31^ Similarly, a study conducted in Canada also reported that the incidence of diabetes increased more over time in PwMS than in age, sex and geographically matched controls, although another study found that PwMS with diabetes had 57% increased odds of achieving a HbA1c of ⩽ 7% as compared to controls with diabetes.^2,5^ The first study also reported that temporal trends in the incidence of hypertension did not differ.^5^ We found sex differences in the risk of vascular comorbidities, especially for Type 2 diabetes, with the relative risk being much greater for women than men. Differences were attenuated for hypertension, although, on a relative scale, women with MS were more likely to commence antihypertensive medication than men with MS, as compared with matched controls. Sex differences in vascular risk in PwMS have been previously documented.^3^ Although women with MS might lose the pre-menopause protection against vascular disease, consistently with evidence regarding the general population, we found that they were more likely to reach targets for blood pressure.^32^

Study strengths include the large study sample, which allowed us to assess overall and sex-related differences in vascular risk management over time. We controlled for significant clinical variables, BMI, blood pressure, and frailty index when assessing these differences. Accounting for frailty was a particularly novel aspect of the study. A significantly higher percentage of PwMS is frail,^9,14^ which is strongly associated with disability measured using the EDSS, disease duration and fatigue.^14,15^ The frailty index is also associated with an increased risk of macrovascular disease and mortality.^11–13^ Additional strengths include the fact that in the United Kingdom, most MS diagnosis and treatment are made by the NHS,^33^ hence the stability of the methods through a longitudinal study across a decade.

Several caveats merit discussion. First, the CPRD includes only a sample of practices, and the database only holds data for prescriptions with no information about whether the medication has been dispensed or adhered to.^16^ Second, when assessing diabetes and cholesterol management, we only considered differences in rates of medication prescription, and it was not possible to look at trajectories of HbA1c, fasting plasma glucose, and cholesterol considering the high percentage of missing for those variables. However, we could analyse differences in trajectories of BP and BMI as we used imputed data on these variables, considering that in previous research on the same study population we showed that results using imputed data were comparable to those obtained from the complete case data.^9^ Third, we could not examine the management of behavioural risk factors for vascular disease, although we controlled for them in our analyses. Fourth, considering that PwMS of non-White ethnic groups represented only 6% of our study population, findings might not be comparable with those from studies conducted on different ethnic groups, such as Latinx/Hispanic, in which untreated hypertension was more likely found than in non-Latinx/Hispanic.^34^ Fifth, we lacked MS-related characteristics, such as relapse rates. Finally, the findings may not be generalised to other Health Systems, but concordance of some findings with those from a Canadian study is reassuring in terms of potential applicability to other publicly funded health systems.^2^

Despite growing interest in the impact of vascular risk management in PwMS, insufficient high-quality information is available regarding the incidence or prevalence of vascular risk factors in PwMS over the disease course. Although the observation that PwMS with hypertension are more likely to meet treatment targets than matched controls might be encouraging, PwMS continue to have increased rates of hypertension and Type 2 diabetes, as compared with people without MS. A possible reason for this discordance is that the vascular guidelines for PwMS are insufficient to adequately control their MS-disease-specific vascular risk factor profile, compared with the general population. The increased vascular burden in PwMS is yet to be fully understood. Biological reasons, including the inflammatory environment that characterise the MS, might partially explain it.^35^ Hence, different and possibly more stringent treatment targets and tailored management tools may be needed to reduce the vascular burden and mitigate the consequence of the disease. This remains to be explored.

## Supplemental Material

sj-docx-1-msj-10.1177_13524585241287388 – Supplemental material for Trajectories and management of vascular risk following the diagnosis of multiple sclerosis: A population-based matched cohort study between 1987 and 2018 in England Supplemental material, sj-docx-1-msj-10.1177_13524585241287388 for Trajectories and management of vascular risk following the diagnosis of multiple sclerosis: A population-based matched cohort study between 1987 and 2018 in England by Raffaele Palladino, Jeremy Chataway, Mekha Mathew, Azeem Majeed and Ruth Ann Marrie in Multiple Sclerosis Journal
