# Supplementary material for: Trajectories and management of vascular risk following the diagnosis of multiple sclerosis: A population-based matched cohort study between 1987 and 2018 in England
Source: Mult Scler. 2024 Oct 17;30(13):1653–63. doi: 10.1177/13524585241287388 (PMC11568649; doi:10.1177/13524585241287388)

**Supplementary Table 1**. Estimated 10-year Incident Rates of Study Outcomes Stratified by MS Status and Sex

| 10-year incident rates (cases x 100,000 person-years | **People with MS** | **Matched Controls** |
| --- | --- | --- |
| **Type 2 diabetes** |  |  |
| Overall | 292.0 (257.9-330.7) | 184.6 (173.5-196.3) |
| Men | 297.9 (236.8-374.7) | 218.5 (196.9-242.5) |
| Women | 289.7 (249.8-335.9) | 170.3 (157.8-183.8) |
| **Hypertension** |  |  |
| Overall | 1433.3 (1352.5-1518.9) | 1210.3 (1180.7-1240.7) |
| Men | 1711.4 (1549.3-1890.5) | 1445.2 (1386.2-1506.7) |
| Women | 1322.7 (1231.6-1420.5) | 1111.5 (1077.7-1146.3) |

**Supplementary Table 2.** Proportion and median time to reach NICE targets for hypertension over the study period for people with MS and matched controls with a diagnosis of hypertension at index year.

| **N** | **People with MS** | **Matched controls** |
| --- | --- | --- |
|  | 1282 | 5552 |
| **Median (years)** | 2 | 3 |
| **Proportion over time (%)** |  |  |
| 0-5 years | 75.0 | 68.3 |
| 0-10 years | 78.9 | 73.6 |
| 0-15 years | 79.8 | 75.1 |
| 0-20 years | 80.0 | 75.5 |
| 0-25 years | 80.0 | 75.5 |

**Supplementary Figure 1.** Cumulative hazards of diagnosis of diabetes for people with Multiple Sclerosis and matched controls in England

Notes: figure A shows results for the entire study population, figure B restricts analysis to men and figure C to women. Index date was defined as the date of the first recorded MS diagnosis event; red line refers to people with Multiple Sclerosis and blue line to matched controls.


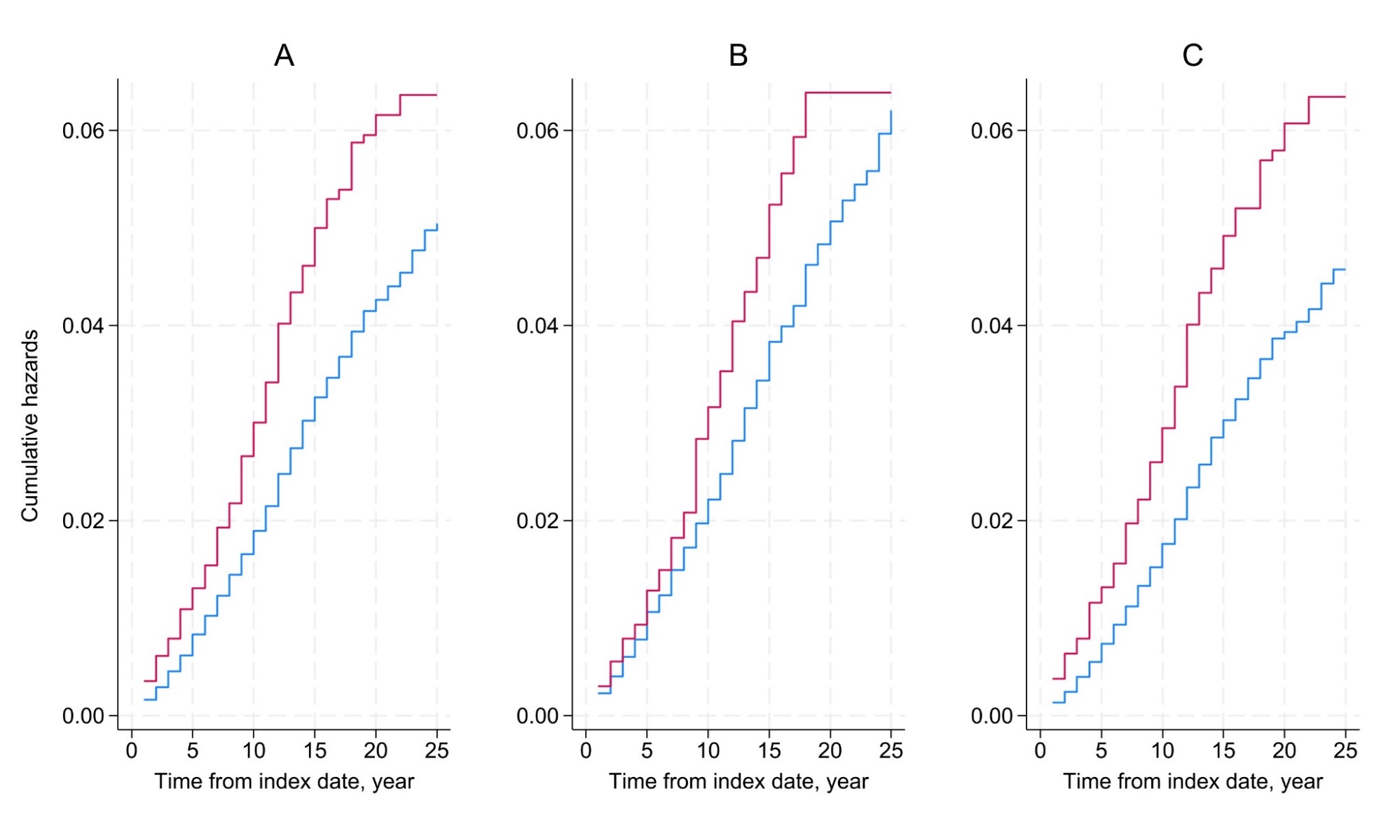


**Supplementary Figure 2.** Cumulative hazards of commencing antidiabetic treatment for people with Multiple Sclerosis and matched controls in England

Notes: figure A shows results for the entire study population, figure B restricts analysis to men and figure C to women. Index date was defined as the date of the first recorded MS diagnosis event; red line refers to people with Multiple Sclerosis and blue line to matched controls.


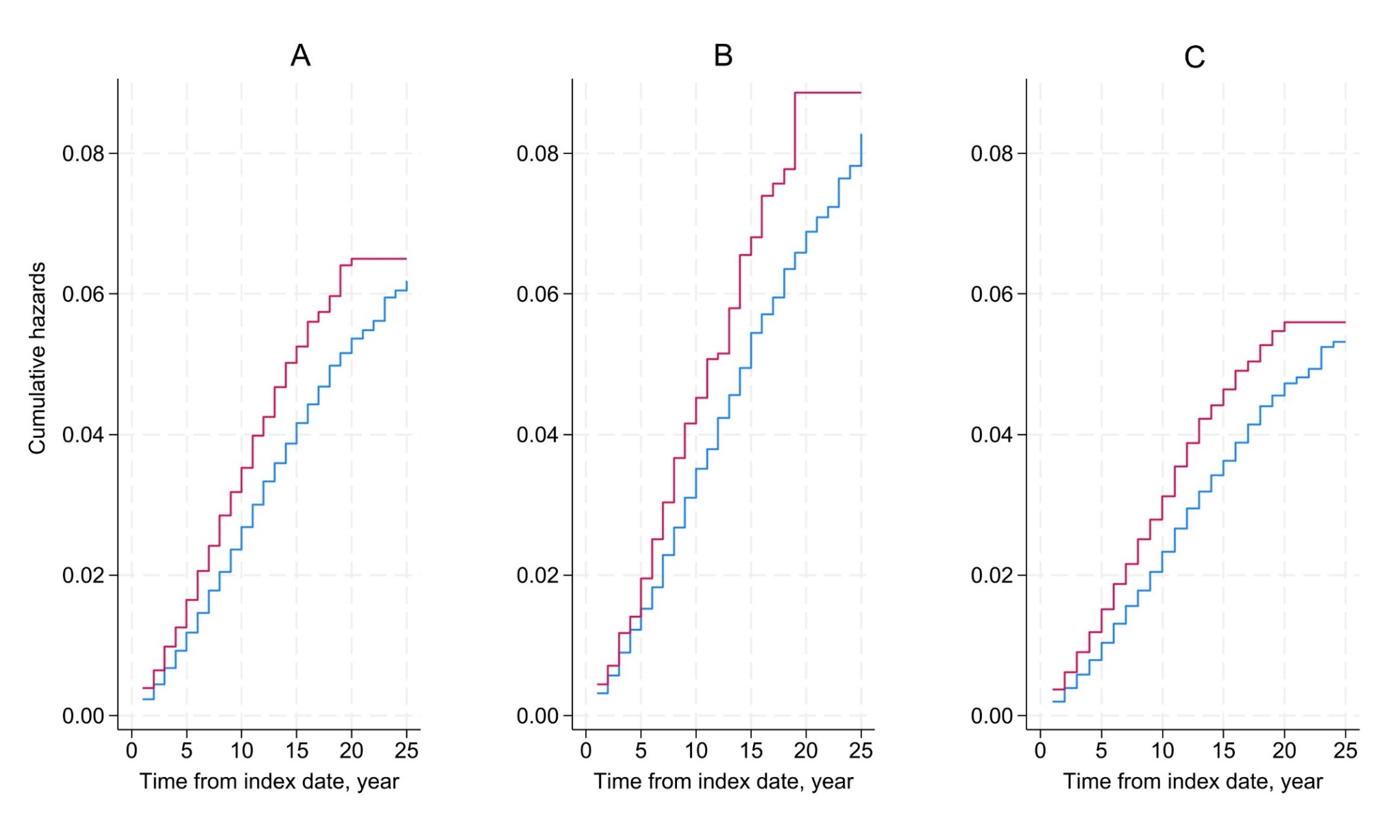


**Supplementary Figure 3.** Cumulative hazards of diagnosis of hypertension for people with Multiple Sclerosis and matched controls in England

Notes: figure A shows results for the entire study population, figure B restricts analysis to men and figure C to women. Index date was defined as the date of the first recorded MS diagnosis event; red line refers to people with Multiple Sclerosis and blue line to matched controls.


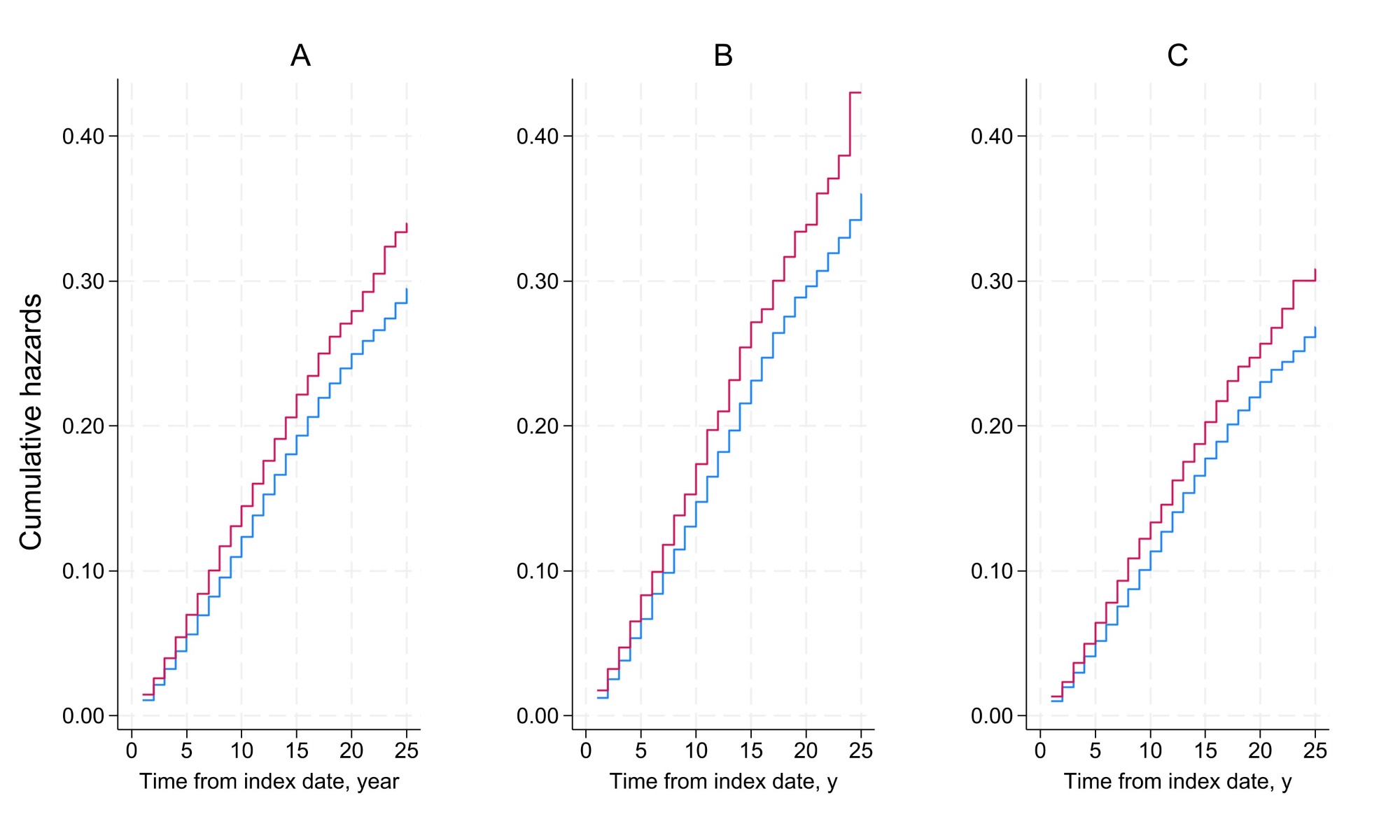


**Supplementary Figure 4.** Cumulative hazards of commencing anti-hypertensive treatment for people with Multiple Sclerosis and matched controls in England

Notes: figure A shows results for the entire study population, figure B restricts analysis to men and figure C to women. index date was defined as the date of the first recorded MS diagnosis event; red line refers to people with Multiple Sclerosis and blue line to matched controls.


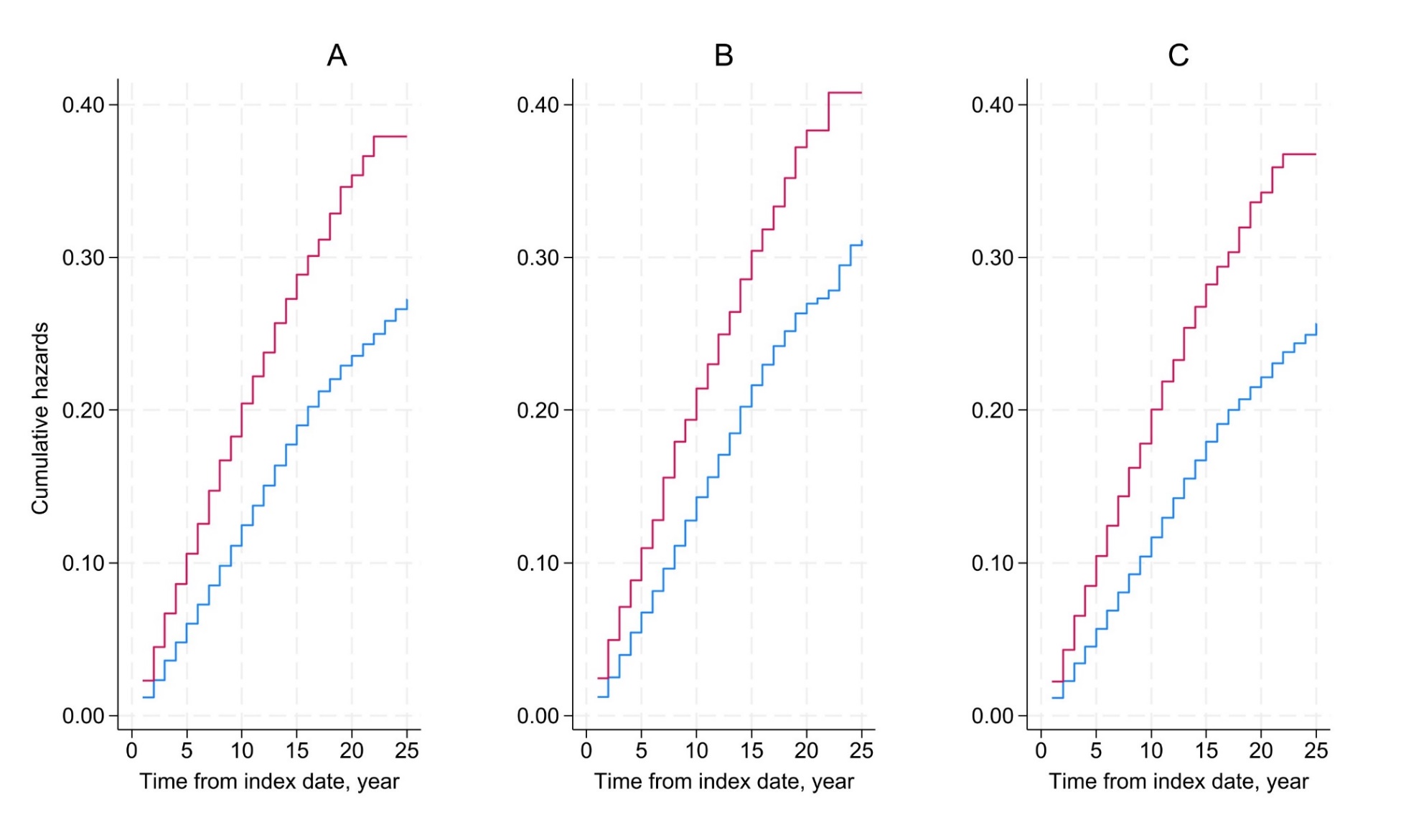


**Supplementary Figure 5.** Cumulative hazards of hitting NICE targets for people with Multiple Sclerosis and matched controls who already have a diagnosis of hypertension at index date in England.

Notes: figure A shows results for the entire study population, figure B restricts analysis to men and figure C to women. Index date was defined as the date of the first recorded MS diagnosis event; red line refers to people with Multiple Sclerosis and blue line to matched controls.


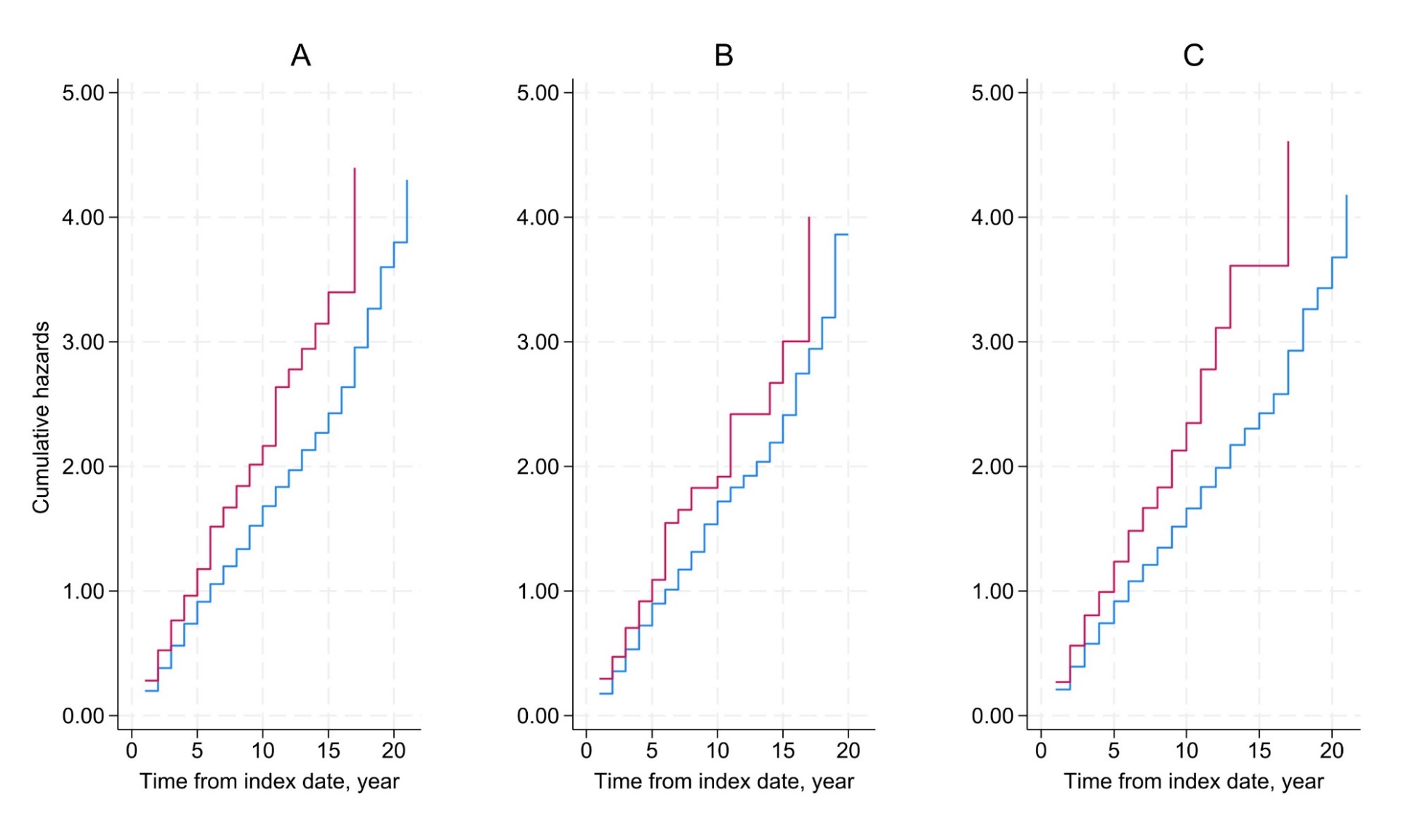


**Supplementary Figure 6.** Cumulative hazards of commencing lipid lowering treatment for people with Multiple Sclerosis and matched controls in England

Notes: figure A shows results for the entire study population, figure B restricts analysis to men and figure C to women. Index date was defined as the date of the first recorded MS diagnosis event; red line refers to people with Multiple Sclerosis and blue line to matched controls.


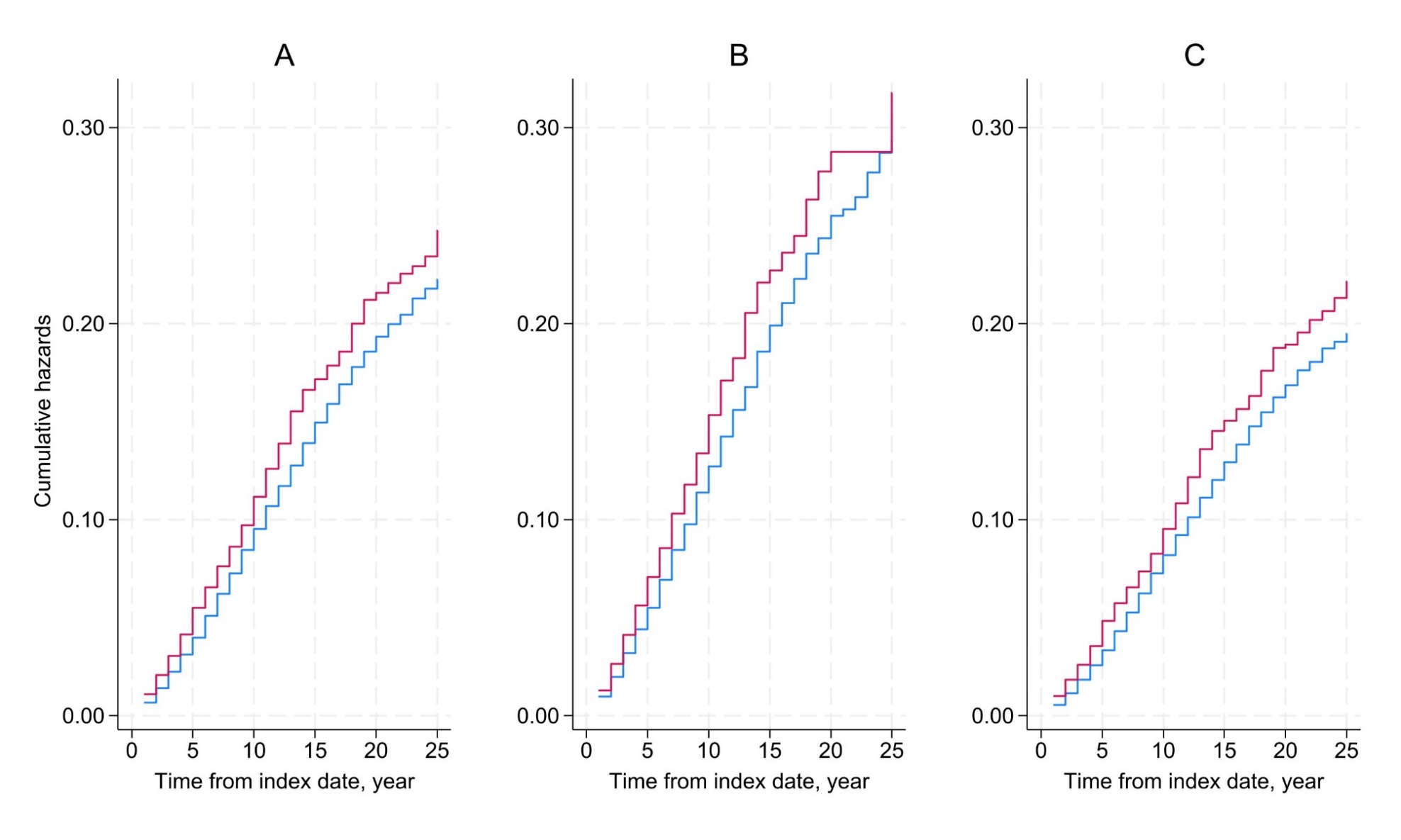

Supplement: sj-docx-1-msj-10.1177_13524585241287388 – Supplemental material for Trajectories and management of vascular risk following the diagnosis of multiple sclerosis: A population-based matched cohort study between 1987 and 2018 in England [file sj-docx-1-msj-10.1177_13524585241287388.docx]
